# Hematopoietic stem cell transplantation therapy for refractory’ Crohn disease: A systematic review and meta-analysis

**DOI:** 10.1097/MD.0000000000040144

**Published:** 2024-10-18

**Authors:** Victor Serrano-Fernandez, Juan Manuel Carmona-Torres, Almudena Arroyo-Rodriguez, Angel Lopez-Gonzalez, Joseba Rabanales-Sotos, Jose Alberto Laredo-Aguilera

**Affiliations:** a Facultad de Fisioterapia y Enfermería, Universidad de Castilla-La Mancha, Toledo, Spain; b Hospital Universitario de Toledo, Toledo, Spain; c Grupo de investigación multidisciplinar en cuidados (IMCU), Universidad de Castilla-La Mancha, Toledo, Spain; d Centro Universitario de Enfermería “San Juan de Dios,” Universidad de Sevilla, Sevilla, Spain; e Facultad de Enfermería, Universidad de Castilla-La Mancha, Albacete, Spain; f Grupo de Actividades Preventivas en el ámbito Universitario de Ciencias de la Salud (GAP-CS), Universidad de Castilla-La Mancha, Albacete, Spain.

**Keywords:** Crohn’s disease, hematopoietic stem cells, inflammatory bowel disease

## Abstract

**Background:**

Despite the availability of numerous treatments for Crohn disease, there are patients who do not respond to any therapy, thereby diminishing their quality of life. The aim of this review is to analyze the efficacy and safety of autologous hematopoietic stem cell transplantation therapy for refractory Crohn disease.

**Methods:**

This work is a systematic review with meta-analysis conducted in accordance with the guidelines of the Preferred Reporting Items for Systematic Review and Meta-Analyses. Electronic databases such as PubMed, Scopus, Web of Science, and ClinicalTrials were consulted. The searches were carried out in August 2024. To evaluate the efficacy of autologous hematopoietic stem cell transplantation in inducing remission, the mean and standard deviation of the Crohn’s Disease Activity Index pre- and post- treatment were used, and a fixed-effects meta-analysis was conducted. Additionally, to assess the efficacy in perianal fistulas, a random-effects meta-analysis was performed, collecting data on the number of subjects with fistulas at the beginning and end of the intervention. All 95% confidence intervals were calculated, and the *I*^2^ statistic was used to assess the heterogeneity of the outcome variables.

**Results:**

A total of 609 records were identified from databases, with 12 studies selected for inclusion in the review. Immediate intervention proved effective in inducing a decrease in the Crohn Disease Activity Index compared to late intervention with conventional therapies. Moreover, the meta-analysis demonstrated efficacy for Crohn disease and associated fistulas with a mean decrease in the CDAI of −217.53 ± 14.3. When evaluating the efficacy of the procedure in perianal fistulas, a risk ratio of 0.47 with a 95% CI of [0.26, 0.86] was obtained. However, the procedure showed adverse effects, such as infections, acute renal failure or deaths.

**Conclusion:**

Systemic autologous hematopoietic stem cell transplantation has shown efficacy in patients who fail to achieve remission of their Crohn disease with conventional therapies. This procedure has also demonstrated efficacy in treating perianal fistulas. However, it is essential to carefully evaluate de implementation of this procedure due to the associated risks.

## 1. Introduction

Crohn disease (CD) is a chronic inflammatory bowel disease (IBD).^[1]^ It can affect any part of the digestive tract, from the mouth to the anus, with patterns of intestinal involvement that alternate between healthy and diseased areas.^[2]^ Its inflammation is associated with the appearance of transmural granulocytic infiltrates.^[3]^ The prevalence of CD is at an epidemiological peak in developed Western countries, with an average prevalence of 322 per 100,000 individuals in Europe, while in North America the prevalence is 319 per 100,000 individuals.^[4,5]^ Some 30% of patients diagnosed with CD are less than 20 years old, this pathology presenting a diagnostic peak in young patients.^[2]^

The main symptoms of CD are diarrhea, abdominal pain, rectal bleeding and weight loss.^[6]^ Specifically, perianal disease phenotype appears in 25% to 55% of patients with CD.^[7,8]^ This complication negatively affects their quality of life since it can be associated with anal pain, fecal incontinence, and a greater number of hospitalizations and surgeries.^[7]^ The conventional treatments for perianal fistulas include antibiotics, surgery, seton drainage, and other approaches.^[9]^ Other extraintestinal manifestations may appear at the systemic level, including joint pain, erythema nodosum, thrombotic events, and kidney stones.^[10,11]^ Symptoms of this pathology greatly lower the quality of life, increasing stress and depressive symptoms in patients with CD.^[12]^

The different therapeutic options include the administration of corticosteroids in the acute phase of the disease, aminosalicylates, immunosuppressants, biological agents, and, ultimately, surgical bowel resection in the medium and long term.^[13]^ To induce remission, corticosteroids are the most effective option.^[14,15]^ To achieve persistent clinical remission, aminosalicylates (sulfasalazine and mesalazine), immunosuppressants (6-mercaptopurine and azathioprine), biological agents (infliximab, adalimumab, vedolizumab, ustekinumab, etc.) and small molecules like Janus Kinase antagonists are used.^[15–17]^ There are cases of refractory CD in which conventional therapies are not effective, so these patients may require surgery to remove the intestinal region affected by CD.^[18]^ Unlike for ulcerative colitis, the other type of IBD, surgery is less effective for refractory CD, as it has a higher recurrence rate after surgery,^[19,20]^ though an adequate intake of fiber in the diet is associated with better remission rates in patients receiving infliximab.^[21]^ It is estimated that 25% of CD patients are refractory to available medical and surgical treatments, compromising their quality of life.^[22]^ In these cases, alternative therapies, such as autologous hematopoietic stem cell transplantation (aHSCT), can be considered to induce and maintain remission.^[20]^ This intervention is often used for the treatment of malignant diseases such as leukemia, multiple myeloma, and lymphoma,^[23]^ though it can also be used for benign immune-mediated diseases.^[23,24]^

Hematopoietic stem cells (HSCs) can be extracted from bone marrow.^[25,26]^ In addition, hematopoietic stem cells can be extracted from the same patient (autologous) or from a compatible donor (allogeneic).^[27,28]^ Hematopoietic stem cells can differentiate into different types of blood cells, allowing them to restore the patient’s immune system.^[29–31]^ However, autologous hematopoietic stem cells have some limitations, as they require harvesting from the patient.^[25,26]^

When performing the autologous procedure, first a mobilization regimen is carried out in which the production of stem cells and their release into the bloodstream are stimulated.^[32]^ These stem cells are subsequently extracted from patients by apheresis.^[33]^ Finally, in CD cases, in the systemic administration of HSC, a non-myeloablative conditioning regimen of cyclophosphamide with anti-thymocyte globulin (ATG) is used in which the patient’s immune cells, are eliminated for later reinfusion of stem cells.^[20,21]^ It is noteworthy that the efficacy of hematopoietic stem cell transplantation hinges on the administration of chemotherapeutic agents during the conditioning phase, with HSC serving as a supportive product for the restoration of blood cells.^[32]^ The allogeneic procedure follows the same steps as the autologous procedure: stem cells are extracted from a compatible donor, and the mobilization and apheresis phases are also carried out from the donor.^[34]^

Both types of stem cells can be used to induce and maintain clinical remission in patients with CD.^[35]^ To induce clinical remission in patients with refractory CD, it is not clear whether autologous or allogeneic procedures are more effective, as allogeneic hematopoietic stem cell transplantation is associated with greater morbidity and mortality.^[36,37]^ Autologous hematopoietic stem cells have been effective at inducing and maintaining remission of refractory CD in several clinical trials, but with multiple side effects.^[38–40]^

To our knowledge, there are no updated systematic reviews that analyze the efficacy and safety of aHSCT for refractory CD or for perianal fistulas. The objective of this systematic review was to analyze the available scientific evidence on the efficacy and safety of systemic aHSCT for the treatment of drug-refractory CD and associated fistulas.

## 2. Methods

### 2.1. Design and information sources

**Figure 1.**
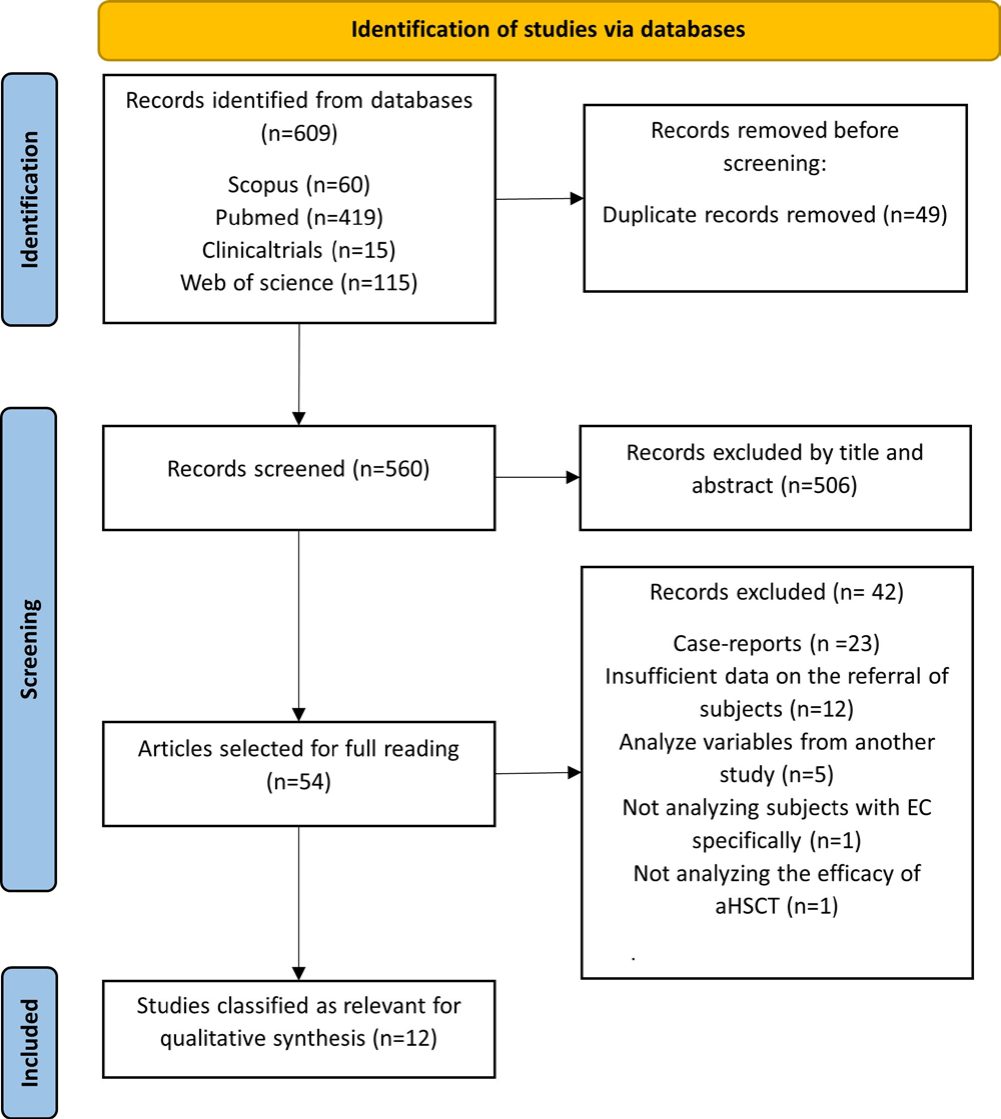
PRISMA flowchart.

This work consists of a systematic review and meta-analysis carried out in accordance with the Preferred Reporting Items for Systematic Reviews and Meta-Analyses (PRISMA) statement.^[41]^ This review was registered in PROSPERO with registration number CRD42023461759. The details of the study selection process are shown in Figure 1.

We performed the searches in the PubMed, ClinicalTrials, Web of Science, and Scopus databases. This study does not require approval from an ethics committee as it is a systematic review of existing literature.

### 2.2. Search strategy

To perform the searches, we applied the Population, Intervention, Control, and Outcome (PICO) framework (Table 1). The searches were carried out in August 2024.

**Table 1:** PICO question.

Population	Intervention	Control	Outcome
Patients with drug-refractory CD with or without associated fistulas	aHSCT	Conventional therapies with or without delayed transplantation	Induction, maintenance of remission, healing of fistulas

aHSCT = Autologous Hematopoietic Stem Cell Transplantation, CD = Crohn disease.

The clinical question was as follows: In patients with drug-refractory CD with or without associated fistulas, is systemic aHSCT effective compared to conventional therapies for inducing and maintaining clinical remission and curing associated fistulas?

To answer this question, the search strategies listed in Table 2 were carried out.

**Table 2:** Detailed searches.

Database	Search string
Web Of Science	((TI=(“Crohn disease”)) AND TI=(“stem cell transplantation”)) NOT TI=(“ulcerative colitis”)
ClinicalTrials	(Crohn Disease) AND (stem Cell Transplantation) NOT (Ulcerative Colitis)
Pubmed	(Crohn disease) AND (stem cell transplantation) NOT (ulcerative colitis)
Scopus	TITLE (“Crohn disease” ) AND TITLE (“stem cell transplantation” ) AND NOT TITLE (“ulcerative colitis” )

### 2.3. Inclusion and exclusion criteria

The inclusion criteria for the studies included in this review were as follows: experimental studies (randomized clinical trials (RCTs) or nonrandomized and quasiexperimental) or observational studies that analyzed the efficacy of systemic aHSCT for the treatment of CD with or without associated fistulas, evaluated the safety of systemic aHSCT in patients with CD, were written in Spanish or English, and were carried out in humans.

The exclusion criteria were as follows: Mesenchymal stem cells (MSCs) applied for CD; pathologies other than CD in their study population; allogeneic HSCs; and animal studies.

### 2.4. Search outcome

The final selection of studies for qualitative synthesis was carried out by 2 researchers, V.S.F. and J.M.C.T., taking into account the inclusion and exclusion criteria mentioned above. The searches in all the databases yielded a total of 609 results (the Mendeley bibliographic reference manager was used to discard duplicate results). The study selection process was carried out as specified in Figure 1 according to the PRISMA guidelines.^[41]^ Once duplicate results were removed, titles and abstracts were read to assess studies that met the objectives of this review. From those, 54 studies were selected, whose data were exhaustively read to determine which studies would be included. Finally, 12 studies were selected for inclusion in the review. In cases of doubt or discrepancy in the selection of studies, a third author (J.A.L.A.) was consulted.

### 2.5. Quality appraisal

To assess the quality of the selected studies and detect biases, the Cochrane RoB-2 tool was used^[42]^ for RCTs, ROBINS-I^[43]^ for nonrandomized trials of interventions, and ROBINS-E^[44]^ for nonrandomized observational studies. The scores for each study are collected in the Supplementary Material: RCTs in Table S1, Supplemental Digital Content, http://links.lww.com/MD/N759 quasiexperimental studies in Table S2, Supplemental Digital Content, http://links.lww.com/MD/N759 and observational studies in Table S3, Supplemental Digital Content, http://links.lww.com/MD/N759

The RoB-2 tool for RCTs^[42]^ consists of 5 domains that assess the risk of bias in the randomization process, the planned interventions, loss of data, measurement of the outcome variables, and the selection of reported results. In the 5 domains, there are 22 items whose answers can be Yes, Probably yes, Probably not, No, Not applicable (N/A) and Not informed (N/I). For each domain, the risk of bias was calculated, and the score was low, high or some concern. The total risk of bias was calculated as follows: if the risk of bias was low in all domains, then the total risk was categorized as Low; if there was a risk of Some concern in any domain, the risk was labeled Some concern; and there was a high risk of bias if the risk was High in any domain or if multiple domains had Some concern.

The ROBINS-I tool^[43]^ for nonrandomized trials of interventions is divided into 7 domains with 34 items. The domains assess the risk of bias in confounding factors; selection of participants, interventions, deviations from the interventions initially proposed, data lost to follow-up, measurement of the outcome variables, and selection of reported results. Each item has 5 possible answers: Yes, Probably yes, No, Probably not, N/A, and N/I. The risk of bias was assessed for each domain from the answers to all the questions. The risk can be classified as low, moderate, serious, critical, or N/I. The total risk was assessed as follows: a low risk of bias if a low score was obtained in all domains; a moderate risk of bias if a moderate score was obtained in any domain; a serious risk of bias if a serious score was obtained in one domain but no others; a critical risk of bias if a critical score was obtained in at least 1 domain; and N/I if there was no clear indication that the study was at serious or critical risk of bias.

Finally, the ROBINS-E tool^[44]^ was used for nonrandomized observational studies. Its 40 items are divided into 7 domains. The following domains are used to assess the risk of bias: confounding factors, exposure, selection of participants for the study or analysis, postintervention period, lost data, measurement of results, and selection of reported results. As in the previous tool, each item has 5 possible answers: Yes, Probably yes, No, Probably not, N/A, and N/I. For each domain, the risk of bias is estimated based on the responses to each item. This risk can be classified as low risk, some concern, high risk, or very high risk. The overall risk of bias for each study was determined based on the domain with the highest bias score.

No study was excluded from the review, as all the studies were of at least moderate quality. The methodological quality was independently reviewed by the authors V.S.F. and J.A.L.A. The interauthor reliability was high, and any disagreements were discussed with A.A.R. until agreement was reached.

### 2.6. Data extraction

Data were extracted by researchers V.S.F. and J.M.C.T. From each study, the following data were collected: first author, year, and country; study design; characteristics of the population: sample size, age, sex, and selection; study intervention; main results: clinical remission and efficacy in perianal fistulas; and conclusions. When interpreting the results of these studies, aHSCT was considered effective when it induced and maintained remission in patients with refractory CD. Remission was defined as a score on the Crohn disease activity index (CDAI) of less than 150 points^[45]^ in conjunction or not with endoscopic criteria evaluated by the simple endoscopic score for Crohn disease (SES-CD).^[46]^

An intervention was considered safe as long as it did not produce adverse effects exclusively attributable to the performance of the transplant.

### 2.7. Synthesis of the data obtained

A narrative synthesis of the selected studies was carried out. Data regarding the activity of refractive CD were analyzed before and after systemic aHSCT for nonrandomized studies, and the efficacy of aHSCT was compared to that of alternative therapies and/or delayed transplantation. Additionally, data regarding the safety of the procedure were collected. These data included deaths attributable to aHSCT or adverse effects derived from the procedure.

When performing a quantitative analysis, a fixed-effects or random effects model was performed due to statistical heterogeneity. A fixed-effects meta-analysis was performed using the inverse variance method. The standard deviation and mean (x) of the CDAI were calculated before and after the intervention for each study included in the meta-analysis. A random-effects meta-analysis was performed for the presence of fistulas before and after aHSCT, for which we used the inverse variance method, and data were collected on the total number of patients with fistulas before and after the intervention.

Statistical heterogeneity was assessed using the *I*^2^ statistic: *I*^2^ ≤ 25%, 26% to 50%, or ≥ 51% was used to define heterogeneity for statistical significance as low, moderate, or high, respectively. Finally, the effect sizes of all included studies were combined to estimate a summary overall effect size, with a 95% confidence interval (CI). A fixed-effects model was used to compare the efficacy of the procedure in inducing remission before versus after surgery, while a random-effects model was used for evaluating the efficacy of the procedure in treating perianal fistulas.

The level of statistical significance was set at 0.05. To test for publication bias, visual inspection of the funnel plot was used for each meta-analysis. Analyses were performed with Cochrane’s RevMan Web software.

## 3. Results

### 3.1. Study characteristics

After conducting the systematic search, 12 studies that met the inclusion criteria were selected for inclusion in the review.^[22,39,40,47–55]^ Among the included studies, 8^[22,40,49–53]^ were considered good-quality, while 4^[39,47,48,55]^ were of moderate quality. We reached a consensus on the methodological quality of each study by applying the RoB-2, ROBINS-E, and ROBINS-I scales, as shown in Tables S1, S2, Supplemental Digital Content, http://links.lww.com/MD/N759 and S3, http://links.lww.com/MD/N759

All the studies chosen were written in English. They were 2 RCT,^[40,55]^ 7 quasiexperimental studies,^[39,47–50,52,54]^ 2 retrospective observational studies,^[22,51]^ and 1 prospective observational study.^[53]^ In the selected studies, the total population included 286 subjects with refractory CD who were recruited and completed follow-up, of whom 80 had perianal fistulas. 41.4% were men and 58.6% women. The age range was 15 to 67 years. All the patients included in the analyzed studies had CD refractory to the drugs commonly used to induce and/or maintain remission of the disease.^[22,39,40,47–55]^ Additionally, 5 studies collected data on the efficacy of the procedure for perianal fistulas.^[22,40,47,48,54]^ The characteristics of the included studies are summarized in Table 3.

**Table 3:** Results table.

Author/year/country	Design	Population	Intervention	Results		Conclusions	Risk of bias
				Clinical remission	Perianal fistulas		
López-García A, et al^[47]^ 2017 Spain	Quasiexperimental study	29 patients with drug-refractory CD, 21 women and 8 men, aged 16–49 years.	aHSCT. 5-year postintervention follow-up.	One year after the aHSCT, 61% of subjects in clinical remission with CDAI < 150 points. Decrease in the average CDAI from 301.4 to 114.8 points in the first year of follow-up. *P* values < .001.	Improvement in 1 of 6 patients at the end of the study.	aHSCT is an effective and feasible therapy in the treatment of drug-refractory CD. However, it will sometimes be necessary to reintroduce conventional therapies.	Low
Jauregui-Amezaga A, et al^[48]^ 2016 Spain	Quasiexperimental study	21 patients with refractory CD, 18 women and 3 men, aged 17–40.	aHSCT. 1-year postintervention follow-up.	At 1 year, 90% of patients were free of surgery due to remission or low inflammatory activity.	Eleven patients suffered from fistulas at the beginning of the study. Eight experienced healing.	The aHSCT is effective, but supportive measures are necessary to ensure the safety of the procedure.	Low
Oyama Y, et al^[39]^ 2005 United states	Quasiexperimental study	12 patients with refractory CD, 6 women and 6 men, aged 15–38.	aHSCT. 1-year postintervention follow-up.	The mean CDAI decreased from 299.5 to 74.7 points after the intervention. Of the total number of patients, 11 maintained a CDAI < 150 points at the end of follow-up.	N/A	aHSCT could be performed safely for refractory CD. However, longer follow-up is necessary.	Low
Hasselblatt P, et al^[49]^ 2012 Germany,	Quasiexperimental study	12 patients with refractory CD, 4 women and 8 men, aged 24–50.	aHSCT. High doses of cyclophosphamide in the conditioning phase. Follow-up of 0.5–10.3 years.	50% of patients achieved a CDAI < 150 points. During a mean follow-up of 6 months, 75% of patients had a CDAI of less than 173 points, and at 9 months, 55% of patients had no ulcers.	N/A	aHSCT is effective and safe in inducing remission in patients with refractory CD. The authors consider it necessary to carry out more RCTs.	Moderate
Ruiz MA, et al^[50]^ 2017 Portugal	Quasiexperimental study	14 patients with refractory CD, 7 men and 7 women, aged 24–50.	aHSCT. Low doses of Cyclophosphamide in the myeloablation phase. Follow-up 1 month after transplantation.	CDAI at the beginning of 281.2 points vs. 95.8 points 30 days after the end of follow-up. Average duration of neutropenia of 4 days post apheresis.	N/A	Less blood toxicity and fewer infections are observed following aHSCT regimen compared to previous studies.	Low
Hawkey CJ, et al^[40]^ 2015 United Kingdom	RCT	45 patients with refractory CD (23 immediate aHSCT vs. 22 late aHSCT): 24 women and 21 men aged 18–50 years.	Immediate vs. late aHSCT in conjunction with conventional therapies. One-year posttransplant follow-up.	CDAI of 166.7 postintervention for immediate transplantation vs. 298.3 for late transplantation (*P* = .019). SES-CD at 3 years for immediate transplantation vs. 7 for late transplantation (*P* value 0.11).	In both groups, 21 subjects began the trial with associated fistulas. At the end of follow-up, 15 improved.	aHSCT did not result in a statistically improvement at 1 year. Also, the procedure was associated with significant toxicity.	Some concerns
Cassinotti A, et al^[52]^ 2008 Italy	Quasiexperimental study	4 patients with refractory CD. 3 men and a woman from 26 to 45.	aHSCT without isolating CD34 + cells on apheresis. Follow-up of 1 year post intervention.	Mean CDAI decreased from 319 to 91 points at 3 months. Mean SES-CD at the beginning of 14 vs. 23 months after the aHSCT.	N/A	aHSCT without selecting CD34 + cells appear to be effective and safe to induce and maintain remission of drug-refractory CD.	Moderate
Hernanz N, et al^[22]^ 2019 Spain	Retrospective observational study	7 patients with refractory CD, 2 men and 5 women, aged 16–43.	aHSCT carried out during 2011–2017. 6-month follow-up.	After 6 months, 3/7 patients achieved clinical and endoscopic remission with associated pharmacological treatment; 2 patients achieved remission without the need for medication; the other 2 patients still had active CD.	Two out of 3 subjects experienced improvement in perianal fistulas.	aHSCT could be a promising option for patients with refractory CD. In some cases, refractory CD can become drug reactive.	Low
Mahmmod N, et al^[53]^ 2019 Holland	Prospective observational study	8 patients with refractory CD, 5 women and 3 men, aged 40–67 years.	aHSCT performed between 2014 and 2017. One-year posttransplant follow-up.	60% of patients reached a CDAI < 150 points at the end of follow-up. In 4/7 patients, no radiological or endoscopic signs of inflammatory activity were found at 1 year of follow-up.	N/A	More than half of the patients obtain a beneficial response to treatment. Also, the use of a less toxic regimen in the mobilization phase could lead to a reduction in the incidence of adverse events.	Low
Brierley CK, et al^[51]^ 2018 United Kingdom	Retrospective observational multicenter study	82 subjects with refractory CD, 52 women and 30 men, aged 20–65.	aHSCT performed from 1996 to 2015. Follow-up from 6 to 174 months.	73% of subjects resumed drug treatment. In these patients, 57% achieved clinical remission by no abdominal pain and normal stool frequency or significant improvement.	N/A	aHSCT is relatively safe and effective in controlling the activity of drug-resistant CD. The authors conclude that more RCTs are needed.	Low
Burt R, et al^[54]^ 2010 United States	Quasiexperimental study	24 patients with CD refractory to anti-TNF, 12 men and 12 women, aged 15–52 years.	aHSCT. 5-year postintervention follow-up	Remission in 91% of subjects at 1 year and 63% at 2 years. At the end of follow-up, 60% of the subjects remained flare-free. The CDAI decreased in 1 year from 200–250 points to less than 100 (*P* < .001).	18 subjects with perianal fistulas at the beginning of the study. At the end, 4 experienced improvements in their fistulas.	Relapses have occurred in patients. However, 70% of subjects achieved treatment-free remissions for as long as 5 years.	Low
Lindsay J, et al^[55]^ 2024 United Kingdom	RCT	22 patients with refractory CD. 13 in intervention group vs 9 in control group. 10 men and 12 women, aged 18–60 years.	aHSCT. 48-weeks postintervention follow-up	CDAI < 150 was achieved in 57% of patients who received the treatment, compared to 17% in the control group. Also, 40% of patients in treatment group achieved a SES-CD of 0, while the percentage was 0% in the control group.	N/A	aHSCT reduced endoscopic disease activity and CDAI. However, frequent adverse effects led to the study’s early termination	Some concerns

aHSCT = Autologous Hematopoietic Stem Cell Transplantation, CD = Crohn disease, CDAI = Crohn disease Activity Index, RCT = randomized controlled trial, SES-CD = Simple Endoscopic Score for Crohn disease, TNF = tumoral necrosis factor.

### 3.2. aHSCT to induce and maintain remission

Of the 12 studies, 11 analyzed the efficacy of systemic aHSCT alone in inducing and maintaining remission in patients with drug-refractory CD.^[22,39,47–55]^ All the studies showed moderate efficacy in inducing and maintaining remission in patients after the procedure and after follow-up.

In several studies, significant decreases in CDAI were observed, from 185.4 to 224.8 points, after the end of follow-up.^[39,47,50,52]^ In one study, the decrease in the CDAI was 186.6 points in 29 patients, (*P* < .001)^[47]^; however, in other studies, the decrease in CDAI was greater, being 224.8 in 12 patients^[39]^ or 278 in 4 patients^[52]^; however, these decreases were not statistically significant. The percentage of patients with a clinical response after follow-up was reported in 7 studies, in which clinical improvements were observed in 43% to 75% of patients undergoing the intervention.^[22,47,49,51,53–55]^ Also, in 2 studies, endoscopic improvements were reported by the SES-CD, with a decrease of 11.5 points^[52]^ and a statistically significant post-aHSCT score of 7.2 points.^[47]^

In one study, 43% of the subjects did not benefit from aHSCT, but their CD became reactive to drugs, yielding a clinical response with conventional therapies.^[22]^

**Figure 2.**
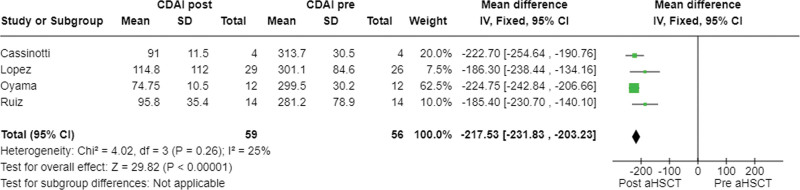
Meta-analysis of CDAI pre- versus post-aHSCT. aHSCT = Autologous Hematopoietic Stem Cell Transplantation, CDAI = Crohn Disease Activity Index.

**Figure 3.**
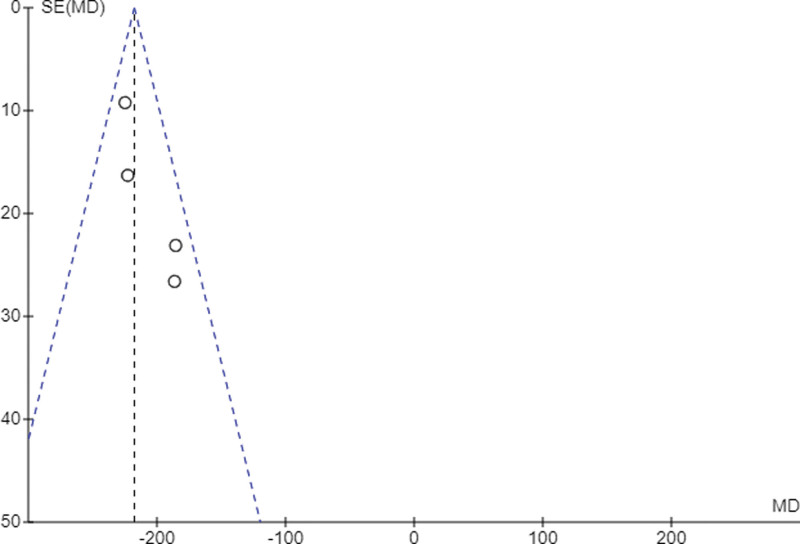
Funnel plot of the CDAI pre- versus post-aHSCT. aHSCT = Autologous Hematopoietic Stem Cell Transplantation, CDAI = Crohn Disease Activity Index.

The results of the meta-analysis can be found in Figure 2. A beneficial effect of the aHSCT was evidenced by a mean decrease in the CDAI of −217.53 ± 14.3, with a low *I*^2^ heterogeneity between studies of 25%. The fixed-effects model was used due to the homogeneity of the outcome variables in the different studies. The risk of publication bias was defined by visual inspection of the funnel plot (Fig. 3).

### 3.3. Immediate aHSCT versus conventional therapies to induce and maintain remission

One study, in which aHSCT was compared with conventional therapies – specifically the one carried out by Hawkey et al^[40]^ performed a comparison between immediate and late transplantations to analyze the effectiveness of the aHSCT. Both groups underwent the mobilization and extraction phase by means of stem cell apheresis and were administered conventional therapies according to their needs. The entire procedure was applied directly to the intervention group, while the transplant was performed 1 year after in the control group. The control group, while waiting for the aHSCT, was administered conventional therapies. After 3 months, the CDAI score decreased by 150.7 points in the patients who received immediate transplantation compared to a decrease of 63 points in the control group; this difference was statistically significant. For the intervention group, the average CDAI and SES-CD at 1 year were 166.7 and 3, respectively, compared to those of the control group, for which the CDAI and SES-CD were 298.3 and 7 points, respectively. However, in this last comparison, only the differences in CDAI were statistically significant.

In another study,^[55]^ patients were divided into 2 groups: those who received aHSCT and those who received conventional therapies. The percentage of patients achieving clinical remission, defined by a CDAI < 150, was 57% in the intervention group compared to 17% in the control group who received standard care. Additionally, 2/5 (40%) of subjects who received systemic aHSCT achieved endoscopic remission defined by a SES-CD ulcer sub-score of 0, while in the control group, this percentage was 0 at the end of the follow-up period.

### 3.4. Efficacy in treating perianal fistulas

Five studies collected data on the evolution of perianal fistulas in patients with refractory CD undergoing systemic aHSCT.^[22,40,47,48,54]^ In total, 80 patients recruited in these studies had perianal fistulas at the beginning of the intervention. In all 5 studies, beneficial data were reported for inducing clinical improvement of fistulas.

In one study, improvements were seen for 6 of 21 patients (28.5%) with this complication.^[40]^ Other studies reported fistula improvement in 1 of 6 patients^[47]^ and 4 out of eighteen patients.^[54]^

Another study reported the evolution of perianal fistulas in patients with refractory CD.^[48]^ Notably, of the 11 patients, only 3 reported worsening of disease; these patients required antibiotic treatment and surgical drainage. In comparison, another study^[22]^ recruited 3 fistula patients from their total sample and observed improvement in 2 patients. A 3-year follow-up was performed in these patients, during which the disappearance of radiological and endoscopic evidence of fistulas was observed.

**Figure 4.**
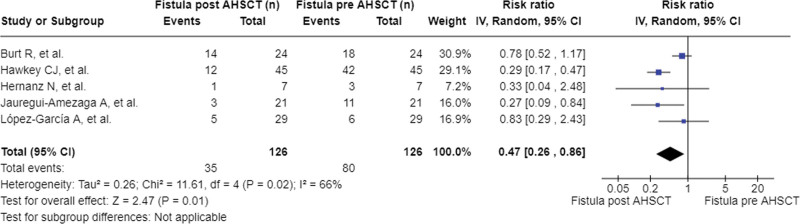
Meta-analysis of fistulas pre- versus post-aHSCT. aHSCT = Autologous Hematopoietic Stem Cell Transplantation, CDAI = Crohn Disease Activity Index.

**Figure 5.**
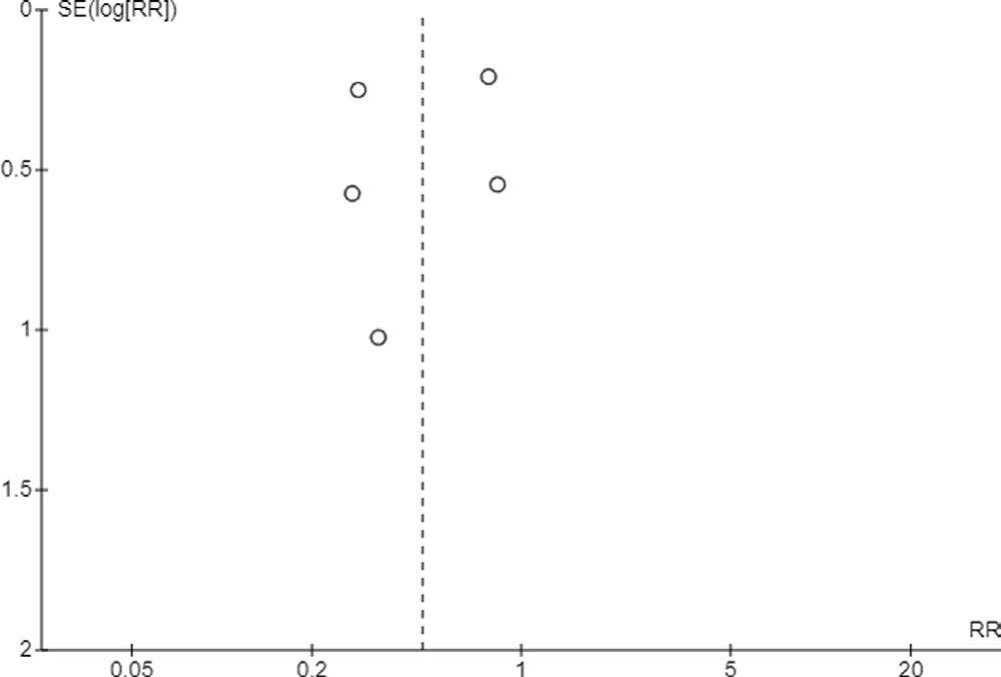
Funnel plot of fistulas pre- versus post-aHSCT. aHSCT = Autologous Hematopoietic Stem Cell Transplantation.

Additionally, a meta-analysis was performed on the efficacy of aHSCT for treating perianal fistulas in patients with refractory CD (Fig. 4). A risk index of 0.47 points was observed when comparing the patients before and after the intervention, with a CI of [0.26, 0.86]. In addition, the random-effects model was used due to the heterogeneity between the outcome variables (I^2^ = 66%). The risk of publication bias was defined by visual inspection of the funnel plot (Fig. 5).

### 3.5. Procedural safety

In all the studies, prophylactic safety measures were applied to the subjects.^[22,39,40,47–55]^ These measures included hospitalization during the mobilization, conditioning, and reinfusion phases.^[39,40,47–52,54,55]^ The authors also conducted analytical surveillance of possible infections^[39,48]^; a diet low in microorganisms^[39,48,54]^; and antibiotic, antiviral and antifungal prophylaxis.^[39,40,48–50,52,54,55]^

In 10 out of the 12 selected studies, adverse reactions caused by aHSCT were described.^[22,40,47–55]^ The most common adverse events were bacterial infections,^[22,40,48–50,54]^ viral infections,^[40,47,48,51,54]^ or fungal infections.^[40,54]^ In 3 studies, the patients had febrile neutropenia.^[22,48,49]^ Other adverse events described include acute renal failure,^[49,55]^ mucositis,^[22,49]^ liver toxicity,^[50]^ pancreatitis,^[50]^ neutropenia,^[50]^ and pancytopenia.^[22,50]^

Also, 4 studies reported deaths during the procedure^[40,48,51,55]^; in two of them, these deaths were due to viral infection caused by cytomegalovirus,^[48,51]^ while other causes included pulmonary veno–occlusive disease^[55]^ and sinusoidal obstructive syndrome as a result of endothelial injury induced by chemotherapy.^[40]^

## 4. Discussion

aHSCT shows moderate efficacy at inducing and maintaining remission in patients with refractory CD who have exhausted conventional therapeutic options.^[22,39,47,49,51–55]^ According to the total sample analyzed in this review, the CDAI score decreased after the intervention, improving the symptoms of the patients.^[39,40,47,49,50,52–54]^ In promoting fistula healing, 2 studies showed great efficacy,^[40,48]^ but in another 3 studies, although there was remission of the fistulas associated with CD, this remission was lower.^[22,47,54]^ Several studies have demonstrated the efficacy of the use of aHSCT for the treatment of other chronic diseases, such as multiple sclerosis, in patients with drug-refractory CD^[56,57]^ or idiopathic arthritis.^[58]^

The regimen consisting of 50 mg/kg/d of cyclophosphamide for 4 days and rabbit ATG at 2.5 mg/kg/d for 3 days was carried out by 6 studies,^[40,47,48,50–52]^ while 2 studies^[39,54]^ used equine ATG at a dose of 30 mg/kg/d for 3 days, and one did not employ any type of ATG.^[49]^ On the other hand, in 1 study,^[53]^ the dose of cyclophosphamide and ATG used in the conditioning regimen was not specified, while another study employed both types of ATG but failed to specify dosages or the allocation of each globulin type among patients.^[22]^

Consistent with the results of this review, several studies that did not meet the inclusion criteria of our review have shown clinical responses in subjects with drug-refractry CD who underwent aHSCT.^[59,60]^ These studies reported clinical improvement in patients once the transplant had been carried out and the follow-up ended. On the other hand, in individual cases in which aHSCT is not effective, the course of CD seems to change because aHSCT reacts to drugs, allowing patients who previously had drug-refractory CD to obtain beneficial responses from these conventional therapies. However, this information was described in only 2 retrospective observational studies,^[22,51]^ so continuing to study this topic is necessary.

In comparison to the use of HSC, infusing allogeneic stem cells as support for blood cell renewal has also shown to have a beneficial effect in a study involving patients with CD.^[61]^ In this study, it was observed that after a 5-year follow-up, most subjects remained in remission according to the CDAI, imaging and endoscopic remission, complete intestinal healing, and histologic remission, with a conditioning regimen of escalating doses of fludarabine and alemtuzumab. It is worth noting that at maximum doses of chemotherapy, 1 patient died, this has also been observed in several of the studies included in this review.^[40,48,51,55]^

The use of stem cells is not the only therapeutic alternative that has emerged in recent years for treating CD. Another alternative procedure that has emerged in recent years is fecal microbiota transplantation to try to induce and maintain remission of CD.^[62,63]^ This therapy is effective because patients with IBD suffer from intestinal dysbiosis, which could be related to the clinical activity of the disease.^[64]^ In this way, by regulating the intestinal microbiota from the feces of healthy donors, clinical improvement is induced in patients.^[65,66]^ However, this therapy has less clear efficacy than aHSCT in inducing and maintaining remission of CD, since several studies conclude that it does not induce significant improvement in these patients.^[67,68]^

Several studies have evaluated the efficacy of stem cells for inducing remission of fistulas associated with CD in patients with perianal fistulas.^[31,69,70]^ However, in contrast with the present review, these studies used MSCs as an intervention and demonstrated that these cells, when administered locally, are both effective and safe in the treatment of perianal fistulas.^[22,31,40,47,48,54,69,70]^ In contrast, the studies included in this review did not focus exclusively on the treatment of perianal phenotypes of CD.^[22,40,47,48,54]^

For inducing and maintaining remission, defined by a decreased CDAI, and for healing fistulas, aHSCT therapy shows efficacy, but with associated adverse events. In contrast, the use of local therapy with MSCs has been more studied and demonstrates great efficacy and safety for the haling of fistula.^[22,39,40,47–54,71–73]^ Regarding obtaining stem cells, there is less complexity in obtaining HSCs since they can be extracted directly from the patient’s blood,^[22,39,40,47–54]^ while MSCs require direct puncture in adipose tissue, bone marrow, or the umbilical cord for subsequent culture.^[31,70,74]^

In general, considering both types of cells, the use of HSCs is the only option for inducing and maintaining remission in CD patients since it improves general symptoms and induces remission in a high percentage of patients, while MSCs have no effect on the systemic level, so they cannot be used to restore the immune system in patients.^[22,31,39,40,47–54,69,70]^ On the other hand, regarding perianal fistulas, it seems that, MSCs are effective when injected locally into the lesion itself, as observed in our review with systemic HSC.^[22,31,40,47,48,54,69,70]^

However, multiple studies have reported adverse effects derived from aHSCT.^[22,40,47–51,53]^ In addition, 4 studies^[40,48,51,55]^ reported deaths attributable to adverse events derived from aHSCT. These findings could be attributed to the nature of the procedure itself, since the study protocols describe how chemotherapeutic agents were used and because the patients who received the intervention were subjected to conditioning regimens.^[22,39,40,47–53,55]^

Due to the high rates of complications, one study^[55]^ have been conducted using lower doses of chemotherapeutic agents during the mobilization phase (cyclophosphamide 1 g/m^2^) and conditioning phase (Fludarabine 25 mg/m^2^, cyclophosphamide 60 mg/kg, and rabbit ATG 2.5 mg/kg). However, it is worth noting that despite reducing pharmacological doses, some patients withdrew from the study, and adverse events were reported in 100% of subjects in the control group, including 1 death.

### 4.1. Limitations and strengths

Among the limitations of this review, it should be noted that only one RCT could be included.^[40]^ In addition, several of the studies included in this review had small samples.^[22,52,53]^ This may be because few patients do not benefit from any conventional therapy, making drug-refractory CD less common in most populations.^[75]^ In addition, although a funnel plot of the results was generated, Egger’s test was not performed since fewer than 10 studies were included in the meta-analyses, making this test underpowered to assess publication bias.^[76,77]^ Although the studies included in both meta-analyses utilized the same doses of cyclophosphamide in the conditioning phase, there is a limitation in some studies which employed equine anti-thymocyte globulin,^[22,39,54]^ unlike the rest of the studies which utilized rabbit anti-thymocyte globulin.^[40,47,48,50,52,55]^

As strengths, in several of the included studies, the clinical status of CD patients was assessed using precise indices such as the CDAI^[39,40,47,49,50,52–55]^ and the SES-CD score.^[47,52,55]^ A similar procedure was used in all interventional studies, and there were only slight modifications in the doses of the drugs used in mobilization and conditioning.^[22,39,40,47–55]^ Two meta-analyses were carried out on the collected studies and their variables.^[22,39,40,47,48,50,52,54]^ Notably, one of the meta-analyses targeted the use of systemic HSCs for the treatment of perianal fistulas.^[22,40,47,48,54]^ This phenomenon has been studied more extensively with MSCs.^[31,69,70,72,73]^ To our knowledge, there are no updated systematic reviews evaluating the efficacy of systemic aHSCT for refractory CD or for associated perianal fistulas.

## 5. Conclusion

aHSCT may be an effective treatment for patients who do not achieve remission of their CD with conventional therapies. However, this procedure is associated with significant adverse effects, including mortality. Therefore, patients with refractory CD should be evaluated individually before initiating the aHSCT due to the risks and costs involved in the procedure. In addition, the correct working of the health care team is essential for minimizing the risk of adverse events and ensuring the effectiveness of the procedure. According to the meta-analysis carried out here, systemic aHSCT are also effective for the treatment of perianal fistulas, although the dearth of studies carried out with hematopoietic cells limits the analysis.

## Author contributions

**Conceptualization:** Victor Serrano-Fernandez, Juan Manuel Carmona-Torres, Almudena Arroyo-Rodriguez, Angel Lopez-Gonzalez, Joseba Rabanales-Sotos, Jose Alberto Laredo-Aguilera.

**Formal analysis:** Victor Serrano-Fernandez, Juan Manuel Carmona-Torres, Joseba Rabanales-Sotos, Jose Alberto Laredo-Aguilera.

**Funding acquisition:** Juan Manuel Carmona-Torres, Angel Lopez-Gonzalez, Joseba Rabanales-Sotos, Jose Alberto Laredo-Aguilera.

**Investigation:** Victor Serrano-Fernandez, Juan Manuel Carmona-Torres, Almudena Arroyo-Rodriguez, Angel Lopez-Gonzalez, Joseba Rabanales-Sotos, Jose Alberto Laredo-Aguilera.

**Methodology:** Victor Serrano-Fernandez, Juan Manuel Carmona-Torres, Almudena Arroyo-Rodriguez, Jose Alberto Laredo-Aguilera.

**Project administration:** Juan Manuel Carmona-Torres, Jose Alberto Laredo-Aguilera.

**Resources:** Victor Serrano-Fernandez, Juan Manuel Carmona-Torres, Almudena Arroyo-Rodriguez, Angel Lopez-Gonzalez, Joseba Rabanales-Sotos, Jose Alberto Laredo-Aguilera.

**Software:** Victor Serrano-Fernandez, Juan Manuel Carmona-Torres, Angel Lopez-Gonzalez, Jose Alberto Laredo-Aguilera.

**Supervision:** Juan Manuel Carmona-Torres, Almudena Arroyo-Rodriguez, Angel Lopez-Gonzalez, Joseba Rabanales-Sotos, Jose Alberto Laredo-Aguilera.

**Visualization:** Victor Serrano-Fernandez, Almudena Arroyo-Rodriguez, Angel Lopez-Gonzalez.

**Writing – original draft:** Victor Serrano-Fernandez, Juan Manuel Carmona-Torres, Joseba Rabanales-Sotos, Jose Alberto Laredo-Aguilera.

**Writing – review & editing:** Juan Manuel Carmona-Torres, Angel Lopez-Gonzalez, Jose Alberto Laredo-Aguilera.

## Supplementary Material


