# Supplementary material for: Hematopoietic stem cell transplantation therapy for refractory’ Crohn disease: A systematic review and meta-analysis
Source: Medicine (Baltimore). 2024 Oct 18;103(42):e40144. doi: 10.1097/MD.0000000000040144 (PMC11495765; doi:10.1097/MD.0000000000040144)
Supplement: Supplementary file 1 [file medi-103-e40144-s001.docx]

**Supplementary Table 1**. Randomized Clinical Trials risk of bias (RoB-2 tool)

| **Domain** | **Signalling questions** | **Hawkey CJ, et al.** | **Lindsay J, et al.** |
| --- | --- | --- | --- |
| Domain 1:  Risk of bias arising from the randomization process | 1.1 Was the allocation sequence random? | Y | Y |
|  | 1.2 Was the allocation sequence concealed until participants were enrolled and assigned to interventions? | Y | Y |
|  | 1.3 Did baseline differences between intervention groups suggest a problem with the randomization process? | N | N |
|  | Risk-of-bias judgement | Low | Low |
| Domain 2: Risk of bias due to deviations from the intended interventions | 2.1. Were participants aware of their assigned intervention during the trial? | Y | Y |
|  | 2.2. Were carers and people delivering the interventions aware of participants' assigned intervention during the trial? | Y | Y |
|  | 2.3. If Y/PY/NI to 2.1 or 2.2: Were there deviations from the intended intervention that arose because of the trial context? | N | N |
|  | 2.4 If Y/PY to 2.3: Were these deviations likely to have affected the outcome? | - | - |
|  | 2.5. If Y/PY/NI to 2.4: Were these deviations from intended intervention balanced between groups? | - | - |
|  | 2.6 Was an appropriate analysis used to estimate the effect of assignment to intervention? | Y | Y |
|  | 2.7 If N/PN/NI to 2.6: Was there potential for a substantial impact (on the result) of the failure to analyse participants in the group to which they were randomized? | - | - |
|  | Risk-of-bias judgement | Low | Low |
| Domain 3: Missing outcome data | 3.1 Were data for this outcome available for all, or nearly all, participants randomized? | Y | N |
|  | 3.2 If N/PN/NI to 3.1: Is there evidence that the result was not biased by missing outcome data? | - | N |
|  | 3.3 If N/PN to 3.2: Could missingness in the outcome depend on its true value? | - | N |
|  | 3.4 If Y/PY/NI to 3.3: Is it likely that missingness in the outcome depended on its true value? | - | - |
|  | Risk-of-bias judgement | Low | Some concerns |
| Domain 4: Risk of bias in measurement of the outcome | 4.1 Was the method of measuring the outcome inappropriate? | N | N |
|  | 4.2 Could measurement or ascertainment of the outcome have differed between intervention groups? | N | N |
|  | 4.3 If N/PN/NI to 4.1 and 4.2: Were outcome assessors aware of the intervention received by study participants? | Y | Y |
|  | 4.4 If Y/PY/NI to 4.3: Could assessment of the outcome have been influenced by knowledge of intervention received? | PN | NI |
|  | 4.5 If Y/PY/NI to 4.4: Is it likely that assessment of the outcome was influenced by knowledge of intervention received? | N | N |
|  | Risk-of-bias judgement | Some concerns | Some concerns |
| Domain 5: Risk of bias in selection of the reported result | 5.1 Were the data that produced this result analysed in accordance with a pre-specified analysis plan that was finalized before unblinded outcome data were available for analysis? | N/I | Y |
|  | Is the numerical result being assessed likely to have been selected, on the basis of the results, from... |  |  |
|  | 5.2. ... multiple eligible outcome measurements (e.g. scales, definitions, time points) within the outcome domain? | PN | PN |
|  | 5.3 ... multiple eligible analyses of the data? | PN | N |
|  | Risk-of-bias judgement | Some concerns | Low |
| Overall risk of bias |  | Some concerns | Some concerns |

**Supplementary Table 2**. The Risk of Bias In Non-randomized Studies – of Interventions (ROBINS-I tool)

| **Domain** | **Signalling questions** | **López-Garcia A, et al.** | **Jauregui-Amezaga A, et al.** | **Oyama Y, et al.** | **Hasselblatt P, et al.** | **Ruiz MA, et al.** | **Cassinotti A, et al.** | **Burt R, et al.** |
| --- | --- | --- | --- | --- | --- | --- | --- | --- |
| Domain 1: Bias due to  confounding | 1.1 Is there potential for confounding of the effect of intervention in this study?  If N/PN to 1.1: Low risk. No need to consider questions below | N | N | N | N | N | N | N |
|  | 1.2. Was the analysis based on splitting participants’ follow up time according to intervention received?  If N/PN, no need to respond 1.3 | N/A | N/A | N/A | N/A | N/A | N/A | N/A |
|  | 1.3. Were intervention discontinuations or switches likely to be related to factors that are prognostic for the outcome?  If Y/PY, no need to respond 1.4-1.6 | N/A | N/A | N/A | N/A | N/A | N/A | N/A |
|  | 1.4. Did the authors use an appropriate analysis method that controlled for all the important confounding domains? | N/A | N/A | N/A | N/A | N/A | N/A | N/A |
|  | 1.5. If Y/PY to 1.4: Were confounding domains that were controlled for measured -validly and reliably by the variables available in this study? | N/A | N/A | N/A | N/A | N/A | N/A | N/A |
|  | 1.6. Did the authors control for any post-intervention variables that could have been affected by the intervention? | N/A | N/A | N/A | N/A | N/A | N/A | N/A |
|  | 1.7. Did the authors use an appropriate analysis method that adjusted for all the important confounding domains and for time-varying confounding? | N/A | N/A | N/A | N/A | N/A | N/A | N/A |
|  | 1.8. If Y/PY to 1.7: Were confounding domains that were adjusted for measured validly and reliably by the variables available in this study? | N/A | N/A | N/A | N/A | N/A | N/A | N/A |
|  | Risk-of-bias judgement | Low | Low | Low | Low | Low | Low | Low |
| Domain 2: Bias in  selection of  participants  into the study | 2.1. Was selection of participants into the study (or into the analysis) based on participant characteristics observed after the start of intervention?  If N/PN to 2.1: no need to respond 2.2 and 2.3 | N | N | N | N | N | N | N |
|  | 2.2. Were the post-intervention variables that influenced selection likely to be associated with intervention? | N/A | N/A | N/A | N/A | N/A | N/A | N/A |
|  | 2.3 If Y/PY to 2.2: Were the post-intervention variables that influenced selection likely to be influenced by the outcome or a cause of the outcome? | N/A | N/A | N/A | N/A | N/A | N/A | N/A |
|  | 2.4. Do start of follow-up and start of intervention coincide for most participants? | N | N | Y | N | Y | N | PN |
|  | 2.5. If Y/PY to 2.2 and 2.3, or N/PN to 2.4: Were adjustment techniques used that are likely to correct for the presence of selection biases? | Y | Y | N/A | N | N/A | N | Y |
|  | Risk of bias judgement | Low | Low | Low | Moderate | Low | Moderate | Low |
| Domain 3: Bias in  classification  of  interventions | 3.1 Were intervention group clearly defined? | Y | Y | Y | Y | Y | Y | Y |
|  | 3.2 Was the information used to define intervention groups recorded at the start of the intervention? | Y | Y | Y | Y | Y | Y | Y |
|  | 3.3 Could classification of intervention status have been affected by knowledge of the outcome or risk of the outcome? | PY | PY | PY | PY | PY | PY | PY |
|  | Risk of bias judgement | Low | Low | Low | Low | Low | Low | Low |
| Domain 4: Bias due to  deviations  from intended  interventions | 4.1. Were there deviations from the intended intervention beyond what would be expected in usual practice? | N | N | N | PN | N | N | N |
|  | 4.2. If Y/PY to 4.1: Were these deviations from intended intervention unbalanced between groups and likely to have affected the outcome? | N/A | N/A | N/A | N/A | N/A | N/A | N/A |
|  | 4.3. Were important co-interventions balanced across intervention groups? | PY | PY | Y | PY | Y | PY | Y |
|  | 4.4. Was the intervention implemented successfully for most participants? | Y | Y | Y | Y | Y | Y | Y |
|  | 4.5. Did study participants adhere to the assigned intervention regimen? | Y | Y | Y | Y | Y | Y | Y |
|  | 4.6. If N/PN to 4.3, 4.4 or 4.5: Was an appropriate analysis used to estimate the effect of starting and adhering to the intervention? | N/A | N/A | N/A | N/A | N/A | N/A | N/A |
|  | Risk of bias judgement | Low | Low | Low | Low | Low | Low | Low |
| Domain 5: Bias due to  missing data | 5.1 Were outcome data available for all, or nearly all, participants? | Y | Y | Y | Y | Y | Y | Y |
|  | 5.2 Were participants excluded due to missing data on intervention status? | N | N | N | Y | N | N | N |
|  | 5.3 Were participants excluded due to missing data on other variables needed for the analysis? | N | N | N | N | N | N | N |
|  | 5.4 If PN/N to 5.1, or Y/PY to 5.2 or 5.3: Are the proportion of participants and reasons for missing data similar across interventions? | N/A | N/A | N/A | Y | N/A | N/A | N/A |
|  | 5.5 If PN/N to 5.1, or Y/PY to 5.2 or 5.3: Is there evidence that results were robust to the presence of missing data? | N/A | N/A | N/A | Y | N/A | N/A | N/A |
|  | Risk of bias judgement | Low | Low | Low | Low | Low | Low | Low |
| Domain 6: Bias in  measurement  of outcomes | 6.1 Could the outcome measure have been influenced by knowledge of the intervention received? | PY | PY | PY | PY | PY | PY | PY |
|  | 6.2 Were outcome assessors aware of the intervention received by study participants? | Y | PY | Y | Y | Y | Y | Y |
|  | 6.3 Were the methods of outcome assessment comparable across intervention groups? | Y | Y | Y | Y | Y | Y | Y |
|  | 6.4 Were any systematic errors in measurement of the outcome related to intervention received? | PN | PN | N | N | N | PN | PN |
|  | Risk of bias judgement | Low | Low | Low | Low | Low | Low | Low |
| Domain 7: Bias in  selection of  the reported  result | Is the reported effect estimate likely to be selected, on the basis of the results, from... |  |  |  |  |  |  |  |
|  | 7.1. ... multiple outcome measurements within the outcome domain? | N | N | N | N | N | N | N |
|  | 7.2 ... multiple analyses of the intervention-outcome relationship? | PN | N | N | N | PN | PN | N |
|  | 7.3 ... different subgroups? | N | N | N | N | N | N | N |
|  | Risk of bias judgement | Low | Low | Low | Low | Low | Low | Low |
| Overall risk of bias |  | Low | Low | Low | Moderate | Low | Moderate | Low |

**Supplementary Table 3**. The Risk Of Bias In Non-randomized Studies – of Exposures (ROBINS-E tool)

| **Domain** | **Signalling questions** | **Hernanz N, et al.** | **Mahmmod N, et al.** | **Brierley C, et al.** |
| --- | --- | --- | --- | --- |
| Domain 1: Risk of bias due to confounding | 1.1 Did the authors control for all the important confounding factors for which this was necessary? | Y | Y | Y |
|  | 1.2 If Y/PY/WN to 1.1: Were confounding factors that were controlled for (and for which control was necessary) measured validly and reliably by the variables available in this study? | Y | Y | Y |
|  | 1.3 If Y/PY/WN to 1.1: Did the authors control for any variables after the start of the exposure period being studied that could have been affected by the exposure? | N | N | N |
|  | 1.4 Did the use of negative controls, or other considerations, suggest serious uncontrolled confounding? | N | N | N |
|  | Risk of bias judgment | Low | Low | Low |
| Domain 2: Risk of bias arising from measurement of the exposure | 2.1 Does the measured exposure well-characterize the exposure metric specified to be of interest in this study? | Y | Y | Y |
|  | 2.2 Was the exposure likely to be measured with error, or misclassified? | N | PN | PN |
|  | 2.3 If SY/WY to 2.2: Could mismeasurement or misclassification of exposure have been differential? | N/A | N/A | N/A |
|  | 2.4. If SY/WY to 2.2 and N/PN/WY to 2.3: Is non-differential measurement error likely to bias the estimated effect of exposure on outcome? | N/A | N/A | N/A |
|  | Risk of bias judgment | Low | Low | Low |
| Domain 3: Risk of bias in selection of participants into the study | 3.1 Did follow-up begin at the start of the exposure window for most participants? | N | N | N |
|  | 3.2 If N/PN to 3.1: Is the effect of exposure likely to be constant over the period of follow up analysed? | Y | Y | Y |
|  | 3.3 Was selection of participants into the study (or into the analysis) based on participant characteristics observed after the start of the exposure window being studied? | N | N | N |
|  | 3.4 If Y/PY to 3.3: Were these characteristics likely to be influenced by exposure or a cause of exposure? | N/A | N/A | N/A |
|  | 3.5 If Y/PY to 3.4: Were these characteristics likely to be influenced by the outcome or a cause of the outcome? | N/A | N/A | N/A |
|  | 3.6 If N/PN to 3.2 or Y/PY to 3.5: Is it likely that the analysis corrected for all of the potential selection biases identified in A and B above? | N/A | N/A | N/A |
|  | 3.7 If N/PN to 3.2 or Y/PY to 3.5: Did sensitivity analyses demonstrate that the likely impact of the potential selection biases identified in A or B above was minimal? | N/A | N/A | N/A |
|  | Risk of bias judgment | Low | Low | Low |
| Domain 4: Risk of bias due to post-exposure interventions | 4.1 Were there post-exposure interventions that were influenced by prior exposure during the follow-up period? | N | N | N |
|  | 4.2 If Y/PY to 4.1: Is it likely that the analysis corrected for the effect of post-exposure interventions that were influenced by prior exposure? | - | - | - |
|  | Risk of bias judgment | Low | Low | Low |
| Domain 5: Risk of bias due to missing data | 5.1 Were complete data on exposure status available for all, or nearly all, participants? | Y | Y | Y |
|  | 5.2 Were complete data on the outcome available for all, or nearly all, participants? | Y | Y | Y |
|  | 5.3 Were complete data on confounding variables available for all, or nearly all, participants? | N/A | N/A | N/A |
|  | 5.4 If N/PN/NI to 5.1, 5.2 or 5.3: Is the result based on a complete case analysis? | N/A | N/A | N/A |
|  | 5.5 If Y/PY/NI in 5.4: Was exclusion from the analysis because of missing data (in exposure, confounders or the outcome) likely to be related to the true value of the outcome? | N/A | N/A | N/A |
|  | 5.6 If N/PN to 5.5: Were all or most predictors of missingness (in exposure, confounders or the outcome) included in the analysis model? | N/A | N/A | N/A |
|  | 5.7 If N/PN to 5.4: Was the analysis based on imputing missing values? | N/A | N/A | N/A |
|  | 5.8 If Y/PY to 5.7: Was imputation performed appropriately? | N/A | N/A | N/A |
|  | 5.9 If N/PN to 5.7: Was an appropriate alternative method used to correct for bias due to missing data? | N/A | N/A | N/A |
|  | 5.10 If PN/N/NI to 5.1, 5.2 or 5.3: Is there evidence that the result was not biased by missing data? | N/A | N/A | N/A |
|  | Risk of bias judgment | Low | Low | Low |
| Domain 6: Risk of bias arising from measurement of the outcome | 6.1 Could measurement or ascertainment of the outcome have differed between exposure groups or levels of exposure? | N | N | PN |
|  | 6.2 Were outcome assessors aware of study participants’ exposure history? | N | N | N |
|  | 6.3 If Y/PY/NI to 6.2: Could assessment of the outcome have been influenced by knowledge of participants’ exposure history? | N/A | N/A | N/A |
|  | Risk of bias judgment | Low | Low | Low |
| Domain 7: Risk of bias in selection of the reported result | 7.1 Was the result reported in accordance with an available, pre-determined analysis plan? | Y | Y | Y |
|  | 7.2 If N/PN/NI to 7.1: Is the reported effect estimate likely to be selected, based on desirability of the magnitude (or statistical significance) of the estimated effect of exposure on outcome, from multiple exposure measurements within the exposure domain? | N/A | N/A | N/A |
|  | 7.3 Is the reported effect estimate likely to be selected, based on desirability of the magnitude (or statistical significance) of the estimated effect of exposure on outcome, from multiple outcome measurements within the outcome domain? | PN | N | PN |
|  | 7.4 Is the reported effect estimate likely to be selected, based on desirability of the magnitude (or statistical significance) of the estimated effect of exposure on outcome, from multiple analyses of the exposure-outcome relationship? | N | PN | PN |
|  | 7.5 Is the reported effect estimate likely to be selected, based on the basis of desirability of the results (e.g. statistical significance), from different subgroups? | N | N | N |
|  | Risk of bias judgment | Low | Low | Low |
| Overall risk of bias |  | Low | Low | Low |
